# Apoptosis inhibitor of macrophage depletion decreased M1 macrophage accumulation and the incidence of cardiac rupture after myocardial infarction in mice

**DOI:** 10.1371/journal.pone.0187894

**Published:** 2017-11-09

**Authors:** Shohei Ishikawa, Takahisa Noma, Hai Ying Fu, Takashi Matsuzaki, Makoto Ishizawa, Kaori Ishikawa, Kazushi Murakami, Naoki Nishimoto, Akira Nishiyama, Tetsuo Minamino

**Affiliations:** 1 Department of Cardiorenal and Cerebrovascular Medicine, Faculty of Medicine, Kagawa University, Kagawa, Japan; 2 Department of Cardiovascular Medicine, Osaka University Graduate School of Medicine, Suita, Osaka, Japan; 3 Clinical Research Support Center, Kagawa University Hospital, Kagawa, Japan; 4 Department of Pharmacology, Faculty of Medicine, Kagawa University, Kagawa, Japan; Max Delbruck Centrum fur Molekulare Medizin Berlin Buch, GERMANY

## Abstract

**Background:**

Cardiac rupture is an important cause of death in the acute phase after myocardial infarction (MI). Macrophages play a pivotal role in cardiac remodeling after MI. Apoptosis inhibitor of macrophage (AIM) is secreted specifically by macrophages and contributes to macrophage accumulation in inflamed tissue by maintaining survival and recruiting macrophages. In this study, we evaluated the role of AIM in macrophage accumulation in the infarcted myocardium and cardiac rupture after MI.

**Methods and results:**

Wild-type (WT) and AIM^‒/‒^ mice underwent permanent left coronary artery ligation and were followed-up for 7 days. Macrophage accumulation and phenotypes (M1 pro-inflammatory macrophage or M2 anti-inflammatory macrophage) were evaluated by immunohistological analysis and RT-PCR. Matrix metalloproteinase (MMP) activity levels were measured by gelatin zymography. The survival rate was significantly higher (81.1% vs. 48.2%, *P*<0.05), and the cardiac rupture rate was significantly lower in AIM^**‒/‒**^ mice than in WT mice (10.8% vs. 31.5%, *P*<0.05). The number of M1 macrophages and the expression levels of M1 markers (iNOS and IL-6) in the infarcted myocardium were significantly lower in AIM^**‒/‒**^ mice than in WT mice. In contrast, there was no difference in the number of M2 macrophages and the expression of M2 markers (Arg-1, CD206 and TGF-β1) between the two groups. The ratio of apoptotic macrophages in the total macrophages was significantly higher in AIM^**‒/‒**^ mice than in WT mice, although MCP-1 expression did not differ between the two groups. MMP-2 and 9 activity levels in the infarcted myocardium were significantly lower in AIM^**‒/‒**^ mice than in WT mice.

**Conclusions:**

These findings suggest that AIM depletion decreases the levels of M1 macrophages, which are a potent source of MMP-2 and 9, in the infarcted myocardium in the acute phase after MI by promoting macrophage apoptosis, and leads to a decrease in the incidence of cardiac rupture and improvements in survival rates.

## Introduction

Acute myocardial infarction (AMI) is a major cause of death in developed nations [1,2]. Current therapies, including percutaneous coronary intervention (PCI) and pharmaceutical treatments, are effective in reducing mortality in patients with AMI. Although AMI-related mortality has decreased to one-third of its previous levels over the past three decades in Japan [3], the mortality of cardiac rupture due to MI remains high (about 30–80%) [4, 5]. Thus, it is a world-wide unmet clinical need to elucidate the mechanisms of cardiac rupture after AMI.

Macrophages play a pivotal role in cardiac remodeling after MI [6]. Although macrophage-induced inflammatory responses are essential for cardiac repair, they also contribute to the development of cardiac rupture [7]. Macrophages are grouped into the following two phenotypes: a pro-inflammatory (M1) and an anti-inflammatory (M2) phenotypes [8]. The main functions of M1 macrophages include phagocytosis of cellular debris at sites of myocardial damage, secretion of inflammatory cytokines and reorganization of tissue matrices by producing metalloproteinases (MMPs) in the acute phase after MI [9, 10]. In contrast, M2 macrophages facilitate resolution of inflammation and regeneration by promoting myofibroblast accumulation, collagen deposition, and angiogenesis [11].

It was reported that macrophage accumulation in the infarcted myocardium might be involved in cardiac rupture [12, 13], whereas M2 macrophages have been reported to have an inhibitory effect on cardiac rupture [14, 15]. Therefore, it is assumed M1 macrophages in the infarcted myocardium might contribute to cardiac rupture. However, the precise roles of M1 and M2 macrophages in the pathogenesis of cardiac rupture have not been fully elucidated.

Apoptosis inhibitor of macrophage (AIM) is a macrophage-specific secreted protein [16]. AIM appears to increase resistance to multiple initiators of apoptosis, including steroids, irradiation, Fas/CD95, and infection [17, 18]. Furthermore, AIM influences M1 macrophage recruitment to inflammatory tissues by facilitating monocyte chemoattractant protein 1 (MCP-1) expression [19, 20].

Therefore, we hypothesized that AIM may contribute to M1 macrophage accumulation in the infarcted myocardium in the acute phase after MI, which may lead to augmentation of inflammatory response and cardiac rupture, by inhibiting macrophage apoptosis and promoting macrophage recruitment.

In this study, we evaluated the role of AIM in cardiac rupture, M1 and M2 macrophage accumulation and MMP activity levels in the infarcted myocardium after MI in mice.

## Materials and methods

### Ethic statement

This study was carried out in strict accordance with the recommendations in the Guide for the Care and Use of Laboratory Animals of the National Institutes of Health. The protocol was approved by the Animal Care and Use Committee for Kagawa University (Permit Number: 125–2). Isoflurane anesthesia was used to reduce the suffering and distress of the mice during any procedure that was potentially painful or stressful.

### Animals

AIM^−/−^ mice were obtained from Toru Miyazaki at Tokyo University [17]. AIM^−/−^ mice were backcrossed with C57BL/6 mice for at least 15 generations before being used for the experiments described herein. Eight- to ten-week-old male AIM^−/−^ and C57BL/6J wild-type (WT) mice were used in the present study.

### Infarct model

MIs were induced in the above mice as described previously [21]. Briefly, the mice were anesthetized with isoflurane, intubated, and put on a mechanical small-animal ventilator. The chest wall was shaved, and a left thoracotomy was performed at the second left intercostal space. The pericardial sac was opened, and the left coronary artery was permanently ligated with a monofilament nylon 8–0 suture at the site of its emergence from the left atrium. Sham-operated mice in both groups underwent the same procedure but did not undergo coronary artery ligation. Blood pressures and pulse rates were measured by the tail-cuff method under 0.5% isoflurane anesthesia before surgery.

### Experimental protocol

Survival analysis was performed in WT (n = 54) and AIM^−/−^ (n = 37) mice. The mice were followed up for 7 days after surgery. The mice health and behavior were observed every day during the experimental procedure and there were no unexpected deaths among these mice. All dead mice were examined for the presence of MI and the cause of death by autopsy. Cardiac rupture was confirmed based on the presence of blood coagulation around the pericardial sac and in the chest cavity, and heart failure was diagnosed based on the presence of lung congestion with pleural effusions. Day 1 was defined as 24–48 hours after surgery, and mice that died within 24 hours after surgery (day 0) and mice that were found to have small infarcts (grossly <50% of the left ventricular circumference at the mid-papillary level) at the time of sacrifice or autopsy were excluded from the data analysis. Furthermore, separate groups of mice were used for echocardiography or hemodynamic analysis. Echocardiography was performed before and 3, 7 days after MI (n = 16–34 per group). Hemodynamic analysis was performed at 3 days after MI or sham surgery (n = 3–7 per group). After these analyses, mice were sacrificed for histopathological analysis including immunoblotting, immunohistochemistry and real-time PCR. All mice were deeply anesthetized by isoflurane and sacrificed by cervical dislocation.

In order to reduce the suffering of mice, we set humane endpoints to decide when to euthanize the mice. Humane endpoints in the study included decreased activity with respiratory distress, inability to remain upright and seizures.

### Echocardiography and hemodynamic analysis

Transthoracic ultrasound cardiography (UCG) was performed using an echocardiographic system (22-MHz linear transducer; LOGIQ e R6 (GE Healthcare, Amersham, UK). Left ventricular end-diastolic and end-systolic diameter (LVDd and LVDs, respectively) were measured in the M-mode at the level of the papillary muscle, and the heart rate was calculated from the RR interval. Fractional shortening (FS) was calculated as FS (%) = 100 × [(LVDd—LVDs)/LVDd]. Hemodynamic analysis was performed using a 1.4-F micromanometer-tipped catheter (Millar Instruments, Houston, TX, USA) as described [22]. Left ventricular systolic pressure (LVSP) and left ventricular end-diastolic pressure (LVEDP) were measured under light isoflurane anesthesia (0.5%). Measurement of these parameters was performed after intraventricular pressure and HR became stable.

### Morphometric analysis

Heart tissue was fixed in formalin, embedded in paraffin, and cut into 2-μm-thick sections. Sections were stained with picrosirius red to determine the infarct size. Infarct size was calculated as infarct circumference dived by LV circumference as described previously [23].

### Western blot analysis

Western blot analysis was performed as described previously [24, 25]. Proteins extracted from the infarcted myocardium were subjected to 8% polyacrylamide gel electrophoresis and then transferred onto polyvinylidene difluoride membranes. The membranes were then probed using primary antibodies against AIM (1:1000, Trans Genic Inc., Fukuoka, Japan), MMP-2 (1:2000, Abcam, Cambridge, UK) and MMP-9 (1:1000, Abcam, Cambridge, UK).

### Histological analysis

Immunohistochemistry analysis was performed as described previously [22]. Briefly, paraffin sections were stained with rat anti-mouse MAC-3 (1:300, BD Biosciences, Franklin Lakes, NJ, USA) for total macrophages and incubated overnight at 4°C. The following day, the sections were incubated with TaKaRa POD conjugate anti-rat for mouse tissue (Takara Bio Inc., Otsu, Japan) in place of a biotin-labeled secondary antibody, and staining was immediately visualized with DAB (Takara Bio Inc., Otsu, Japan). The number of macrophages was assessed by counting the number of MAC-3-positive cells in the infarcted myocardium at 3 days after MI. To evaluate the phenotype of macrophages, immunofluorescence analysis was performed as described previously [26]. Staining with rabbit anti-mouse iNOS antibody (1:25, Abcam, Cambridge, UK) for M1 macrophages and rabbit anti-mouse CD206 (1:1000, Abcam, Cambridge, UK) for M2 macrophages was performed followed by visualization with anti-rabbit IgG Alexa Fluor 555 (1:1000 Cell signaling, Danvers, MA, USA). Nuclei were stained with mounting medium containing the DAPI fluorescent dye (Wako Pure Chemical Industries Ltd., Osaka, Japan). Apoptotic cells in the infarcted myocardium were detected by Tdt-mediated dUTP nick-end labeling (TUNEL) after proteinase K treatment using the apoptosis detection kit (Takara Bio Inc., Otsu, Japan). Apoptosis of macrophages was evaluated by using mirror sections as described previously [26].

### Real-time PCR

Gene expression was evaluated in the infarct zones of hearts collected at 3 days after MI or sham surgery. Real-time quantitative PCR was performed as described previously [27]. The specific primers used in the present study are shown in Table 1. The relative expression levels of each gene were normalized to Hprt [28] and were quantified using the ΔCT (Ct_Target_−Ct_Hprt_) method, and the fold changes in gene expression compared with sham-operated WT mice were quantified using the 2^-ΔΔCT^ method [29].

**Table 1:** Primers used for real-time PCR in this study.

Gene		Sequence
IL-6	forward	TGATGGATGCTACCAAACTGG
	reverse	TTCATGTACTCCAGGTAGCTATGG
iNOS	forward	TGGCCACCAAGCTGAACT
	reverse	TTCATGATAACGTTTCTGGCTCT
IL-1β	forward	AGTTGACGGACCCCAAAAG
	reverse	AGCTGGATGCTCTCATCAGG
CD206	forward	CCACAGCATTGAGGAGTTTG
	reverse	ACAGCTCATCATTTGGCTCA
Arg-1	forward	GAATCTGCATGGGCAACC
	reverse	GAATCCTGGTACATCTGGGAAC
TGF-β1	forward	TGGAGCAACATGTGGAACTC
	reverse	GTCAGCAGCCGGTTACCA
MCP-1	forward	CATCCACGTGTTGGCTCA
	reverse	GATCATCTTGCTGGTGAATGAGT
Hprt	forward	TCCTCCTCAGACCGCTTTT
	reverse	AACCTGGTTCATCATCGCTAA

iNOS, inducible NO synthase; Arg-1, arginase-1; TGF-β1, transforming growth factor-β1; MCP-1, monocyte chemoattractant protein-1; Hprt, hypoxanthine phosphoribosyltransferase.

### Gelatin zymography

MMP-2 and 9 activity levels in the infarcted myocardium were measured by gelatin zymography at 7 days after MI. Zymography was performed as described previously [30]. Briefly, equal amounts (10 μg) of protein were loaded onto each lane of a 0.15% gelatin zymogram gel. After electrophoresis, the gel was incubated with developing buffer (50 mM Tris-HCl; 200 mM NaCl; and 5 mM CaCl_2_, pH 7.5) for 48 hours at 37°C and stained with Coomassie blue (Bio Rad, USA). Gelatinolytic band intensity was quantified using ImageJ software (Wayne Rasband, National Institutes of Health, USA).

### Statistical analysis

All data are expressed as the mean ± SEM. Survival analysis was performed using the Kaplan-Meier method, and survival curves were compared using the log-rank test. The significance of the differences in the incidence of cardiac rupture between AIM^**‒/‒**^ and WT mice after MI was assessed by Fisher’s exact test. Comparisons between 2 groups were performed with Student’s *t* test (for normally distributed data) or the Mann-Whitney U test (for non-normally distributed data). When more than 2 groups were analyzed, one-way ANOVA with Bonferroni post-hoc test was used. *P*<0.05 was considered significant. All statistical analyses were performed with the SPSS 21.0 statistical software package for Windows (SPSS Inc, Chicago, IL, USA).

## Results

### Cardiac rupture and the infarct size in WT and AIM^‒/‒^ mice after MI

**Fig 1:**
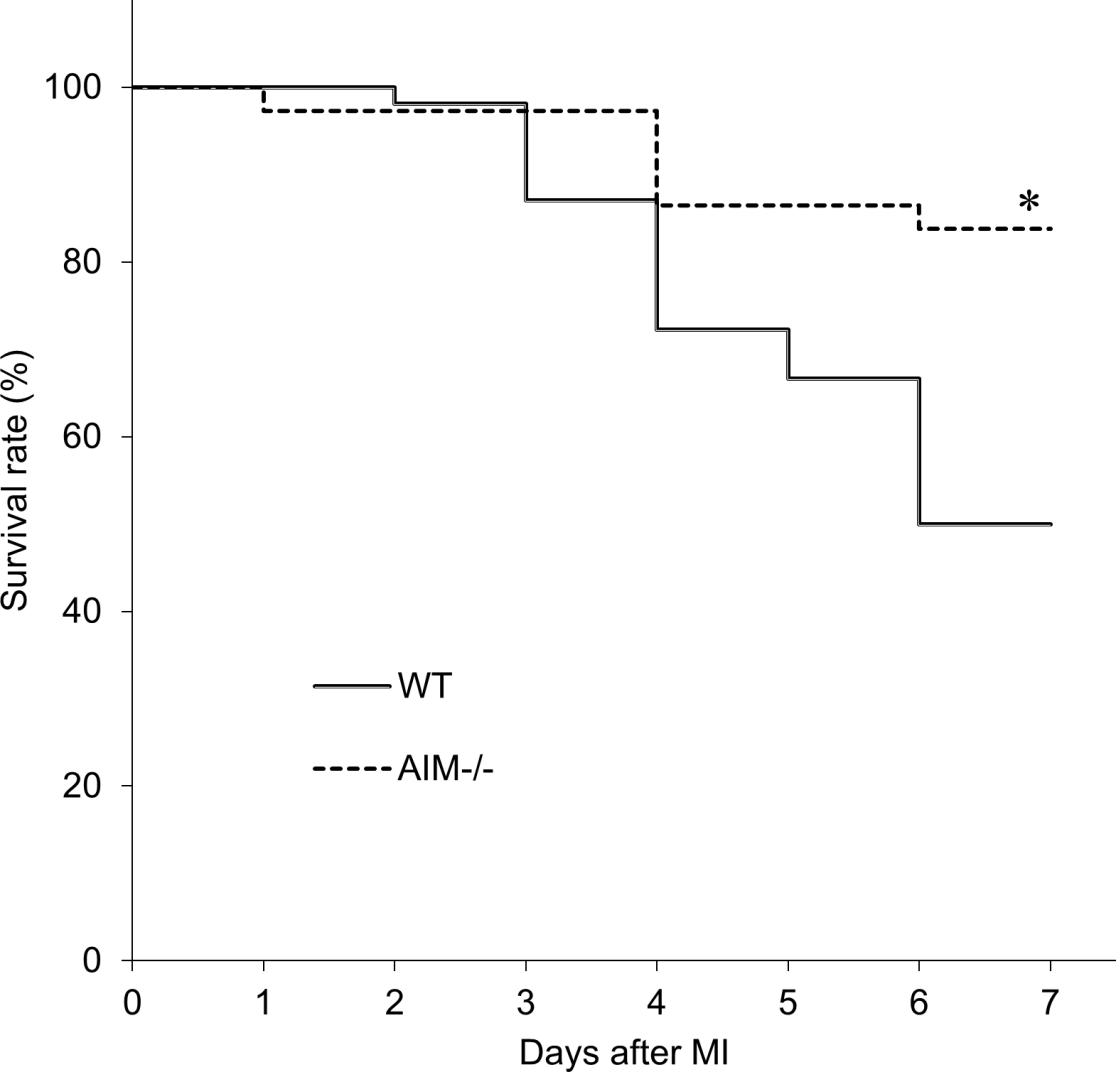
Survival rates for the WT and AIM^‒/‒^ groups after MI. Kaplan-Meier survival curves for the WT and AIM^**‒/‒**^ groups after MI. *P<0.05 compared with WT mice.

**Fig 2:**
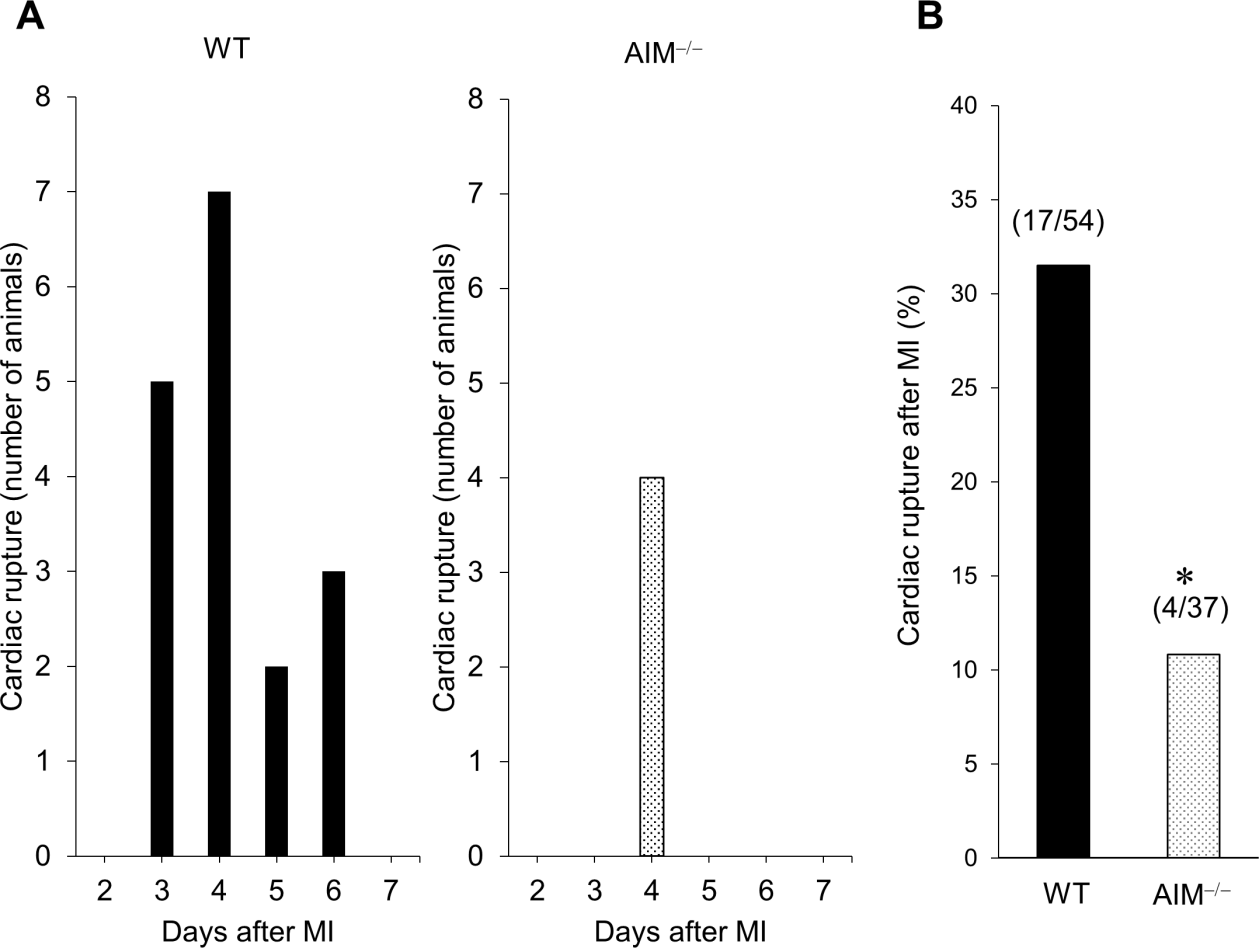
Cardiac rupture in WT and AIM^‒/‒^ mice after MI. The number of animals that died of cardiac rupture in the WT and AIM^**‒/‒**^ groups **(A)**, and the percentages of mice in each group that suffered cardiac rupture after MI **(B)**. *P<0.05 compared with WT mice.

AIM protein was detected in the infarcted myocardium of WT mice, but not AIM^**‒/‒**^ mice (S1 Fig). Compared to the sham-operated myocardium, AIM protein levels increased in the infarcted myocardium in WT mice. Fifty-four WT mice and 37 AIM^**‒/‒**^ mice were used in this survival rate study and 26 WT mice and 30 AIM^**‒/‒**^ mice survived to terminal. AIM^**‒/‒**^ mice had a significantly higher 7-day post-MI survival rate than WT mice (81.1% vs. 48.2%, *P*<0.05; Fig 1). Seventeen WT mice and four AIM^**‒/‒**^ mice suffered fatal rupture of the left ventricular wall between 3 and 6 days after MI (Fig 2A) and the occurrence of cardiac rupture peaked at 4 days after MI in both groups. The rate of cardiac rupture-associated mortality within 6 days after MI was significantly higher in WT mice than in AIM^**‒/‒**^ mice (31.5% vs. 10.8%, *P*<0.05; Fig 2B).

The infarct size determined by the morphometric analysis of picrosirius red stained left ventricular cross-sections at 7 days after MI was comparable (65.7 ± 1.6% vs. 69.2 ± 2.3%, *P* = ns (not significant)) between WT and AIM^**‒/‒**^ mice (S2 Fig).

### Echocardiographic and hemodynamic data for the WT and AIM^‒/‒^ groups before and 3, 7 days after MI

Before coronary artery ligation, there were no significant differences in systolic blood pressure (93.4 mmHg vs. 92.8 mmHg, *P* = ns) or heart rate (558 bpm vs. 563 bpm, *P* = ns), echocardiographic parameters such as LVDd, LVDs and FS between the WT and AIM^**‒/‒**^ groups.

Following MI, LVDd and LVDs were increased, and FS was decreased in WT and AIM^**‒/‒**^ mice. However, there were no significant differences in these parameters between WT and AIM^**‒/‒**^ mice (Table 2). LVSP and LVEDP measured by hemodynamic analysis at 3 days after MI were also comparable between WT and AIM^**‒/‒**^ groups (Table 3).

**Table 2:** Echocardiographic data for the WT and AIM^‒/‒^ groups before and 3, 7 days after MI.

	Before MI		3 days after MI		7 days after MI	
	WT (n = 31)	AIM^‒/‒^ (n = 34)	WT (n = 17)	AIM^‒/‒^ (n = 18)	WT (n = 16)	AIM^‒/‒^ (n = 17)
HR (bpm)	431.1±8.5	444.5±6.1	492.2±11.0	490.0±7.9	485.2±11.0	477.6±7.8
LVDd (mm)	3.37±0.03	3.47±0.05	4.81±0.07*	4.99±0.06*	5.82±0.12*	5.55±0.07*
LVDs (mm)	1.73±0.03	1.80±0.04	4.33±0.08*	4.47±0.09*	5.34±0.14*	5.00±0.10*
FS (%)	48.6±0.8	48.2±0.7	10.1±0.7*	10.4±0.7*	8.3±0.8*	10.1±0.8*

LVDd, left ventricular end-diastolic diameter; LVDs, left ventricular end-systolic diameter; FS, Fractional shortening. Values are means ± SEM.

*P<0.05 compared with the WT mice before MI.

**Table 3:** Hemodynamic data for the WT and AIM^‒/‒^ groups at 3 days after MI.

	Sham		MI	
	WT (n = 3)	AIM^‒/‒^ (n = 3)	WT (n = 5)	AIM^‒/‒^ (n = 7)
Heart rate (bpm)	400.7±3.3	400.7±6.5	401.7±8.9	391.1±7.5
LVSP (mmHg)	105.6±5.4	108.4±5.4	89.4±3.9	89.3±3.4
LVEDP (mmHg)	5.2±0.7	5.8±0.7	12.9±0.9*	13.7±1.1*

LVSP, left ventricular systolic pressure; LVEDP, left ventricular end-diastolic pressure. Values are means ± SEM.

*P<0.05 compared with sham-operated WT mice.

### M1 and M2 macrophages in the infarcted myocardium of WT and AIM^‒/‒^ mice

**Fig 3:**
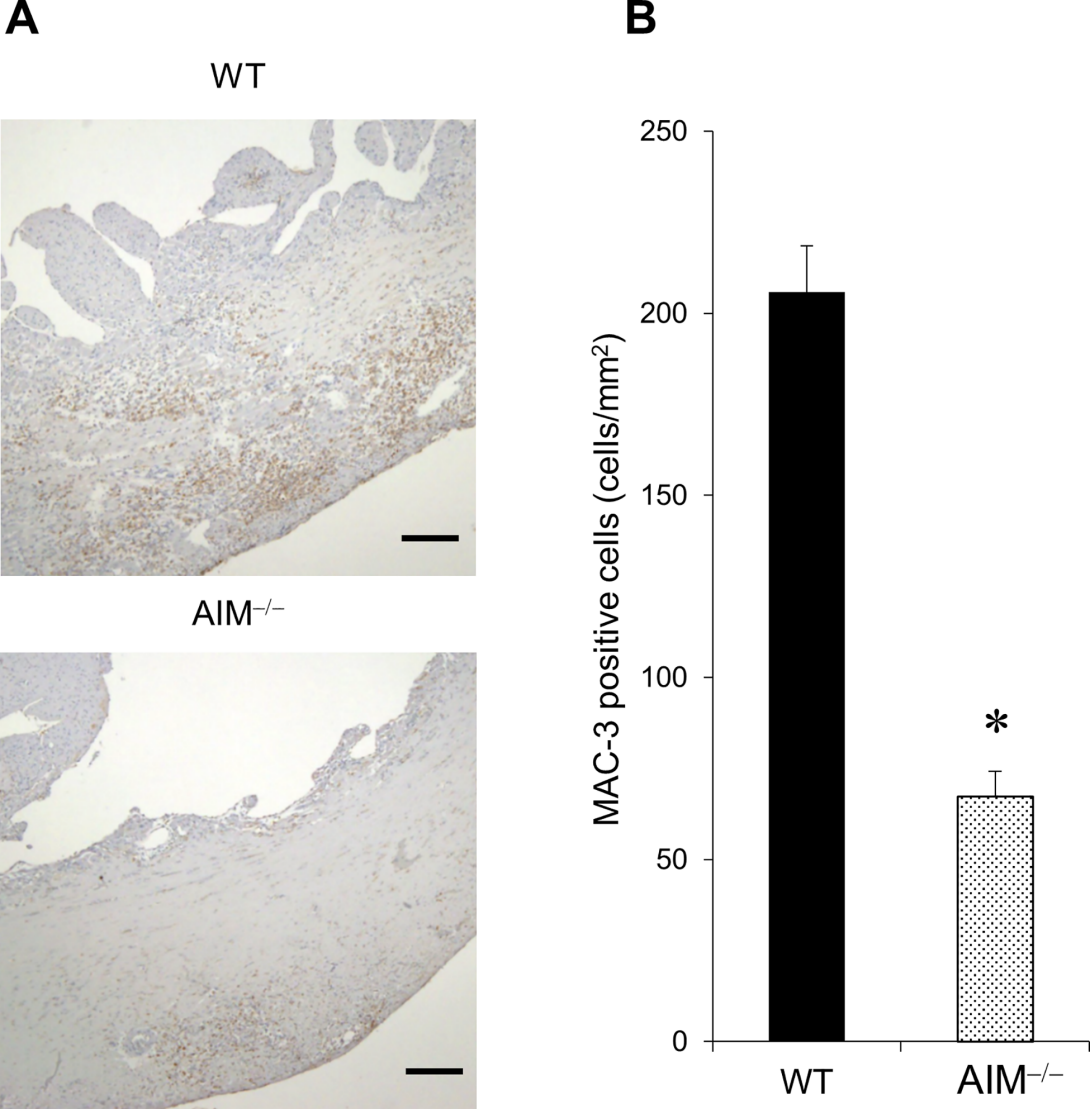
Macrophage accumulation in the infarcted myocardium of WT and AIM^‒/‒^ mice. Representative images of immunohistochemical staining for MAC-3 positive cells in the infarcted myocardium of WT and AIM^**‒/‒**^ mice at 3 days after MI **(A)**. The scale bars indicate 200 μm. The number of MAC-3 positive cells in the infarcted myocardium of WT and AIM^**‒/‒**^ mice at 3 days after MI **(B)**. *P<0.05 compared with WT mice.

We evaluated macrophage accumulation in the infarcted myocardium after MI in WT and AIM^**‒/‒**^ mice by immunohistology and RT-PCR. The number of MAC-3 positive cells indicating total macrophages in the infarcted myocardium was significantly lower in AIM^**‒/‒**^ mice than in WT mice at 3 days after MI (Fig 3A and 3B).

**Fig 4:**
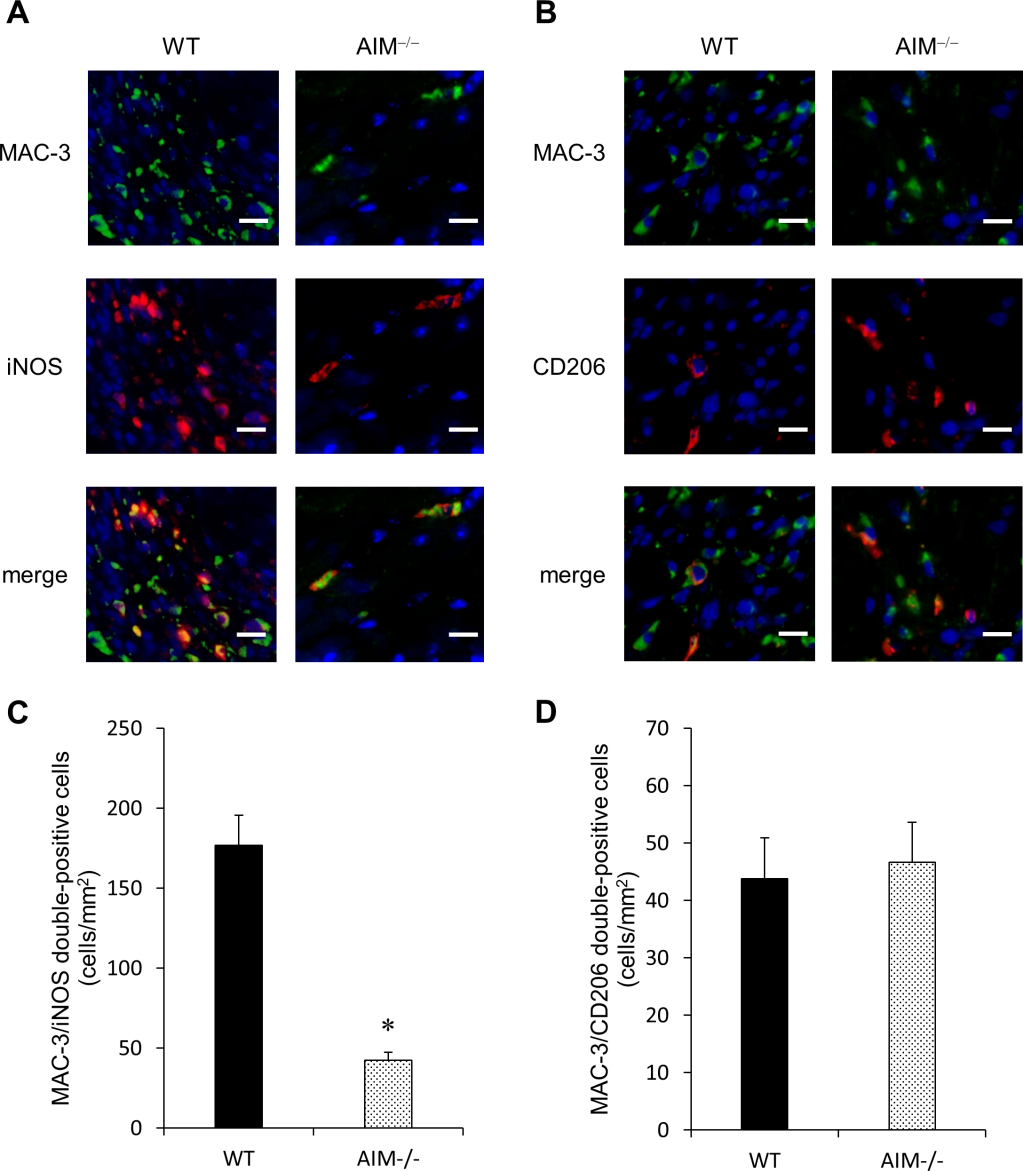
M1 and M2 macrophages in the infarcted myocardium of WT and AIM^‒/‒^ mice at 3 days after MI. Representative images of immunofluorescence staining for MAC-3, iNOS (**A**) and CD206 (**B**). The number of MAC-3/iNOS double-positive cells (**C**) and MAC-3/CD206 double-positive cells (**D**) in the infarcted myocardium. *P<0.05 compared with WT mice.

**Fig 5:**
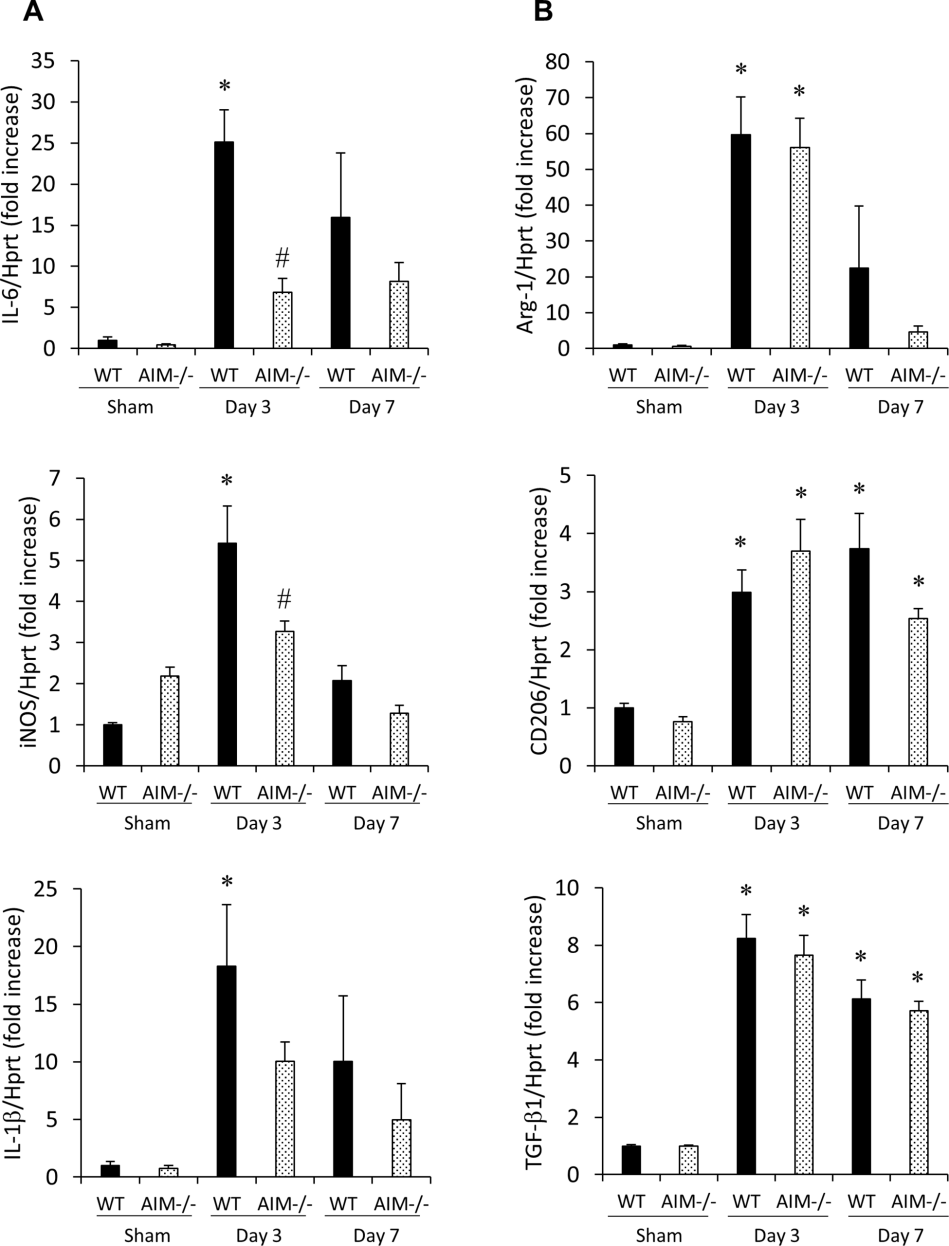
M1 and M2 macrophage marker expression levels in the infarcted myocardium of WT and AIM^‒/‒^ mice. The mRNA levels of the indicated M1 (iNOS, IL-6 and IL-1β; **A**) and M2 (Arg-1, CD206 and TGF-β1; **B**) macrophage markers in the infarcted myocardium of WT and AIM^**‒/‒**^ mice at 3 and 7 days after MI. *P<0.05 compared with sham-operated WT mice, ^#^*P*<0.05 compared with WT-MI mice, n = 7 per group.

Furthermore, we evaluated the number of M1 and M2 macrophages in the infarcted myocardium of WT and AIM^**‒/‒**^ mice at 3 days after MI by double-staining immunofluorescence (Fig 4). The number of MAC-3/iNOS double-positive cells indicating M1 macrophages in the infarcted myocardium was significantly lower in AIM^**‒/‒**^ mice than in WT mice (Fig 4A and 4B). On the other hand, there was no significant difference in the number of MAC-3/CD206 double-positive cells indicating M2 macrophages between the two groups (Fig 4C and 4D). The mRNA levels of the indicated M1 macrophage markers (iNOS and IL-6) in the infarcted myocardium were significantly lower in AIM^**‒/‒**^ mice than in WT mice at 3 days after MI (Fig 5A). The mRNA levels of IL-1β was also tended to be lower in AIM^**‒/‒**^ mice. On the other hand, there was no significant difference in the mRNA levels of the indicated M2 macrophage markers (Arg-1, CD206 and TGF-β1) between WT and AIM^**‒/‒**^ mice (Fig 5B). The mRNA levels of M1 and M2 macrophage markers in the infarcted myocardium at 7 days after MI were not significantly difference between WT and AIM^**‒/‒**^ mice (Fig 5A and 5B).

### Macrophage apoptosis in the infarcted myocardium of WT and AIM^‒/‒^ mice at 3 days after MI

**Fig 6:**
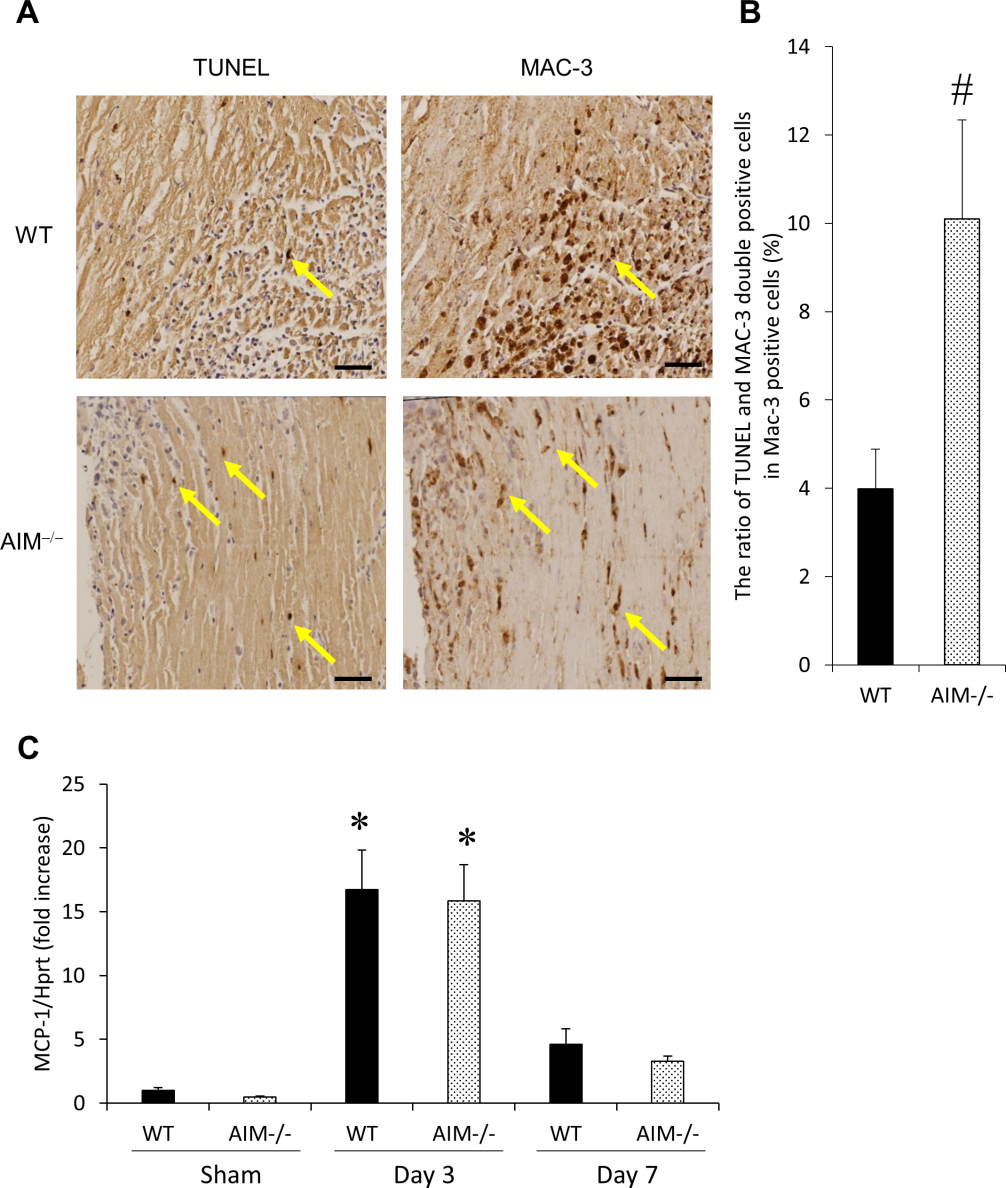
Macrophage apoptosis in the infarcted myocardium of WT and AIM^‒/‒^ mice at 3 days after MI. Representative images of TUNEL/MAC-3 double-positive cells in the infarcted myocardium of WT and AIM^**‒/‒**^ mice at 3 days after MI **(A)**. Mirror sections were stained for TUNEL and MAC-3, respectively and yellow arrows indicate TUNEL/MAC-3 double-positive cells. The ratio of TUNEL/MAC-3 double-positive cells in total MAC-3 positive cells in the infarcted myocardium of WT and AIM^**‒/‒**^ mice at 3 days after MI **(B)**. The mRNA levels of MCP-1 in the infarcted myocardium of WT and AIM^**‒/‒**^ mice at 3 and 7 days after MI (**C**). *P<0.05 compared with sham-operated WT mice, ^#^*P*<0.05 compared with WT-MI mice. n = 6–7 per group.

We evaluated that macrophage apoptosis in the infarcted myocardium of WT and AIM^**‒/‒**^ mice at 3 days after MI (Fig 6A and 6B). The ratio of TUNEL/MAC-3 double-positive cells indicating apoptotic macrophages in total macrophages was significantly higher in AIM^**‒/‒**^ mice than in WT mice.

On the other hand, there was no significant difference in the expression of MCP-1 in the infarcted myocardium at 3 days after MI between WT and AIM^**‒/‒**^ mice (Fig 6C).

### MMP-2 and 9 activity levels in the infarcted myocardium of WT and AIM^‒/‒^ mice at 7 days after MI

**Fig 7:**
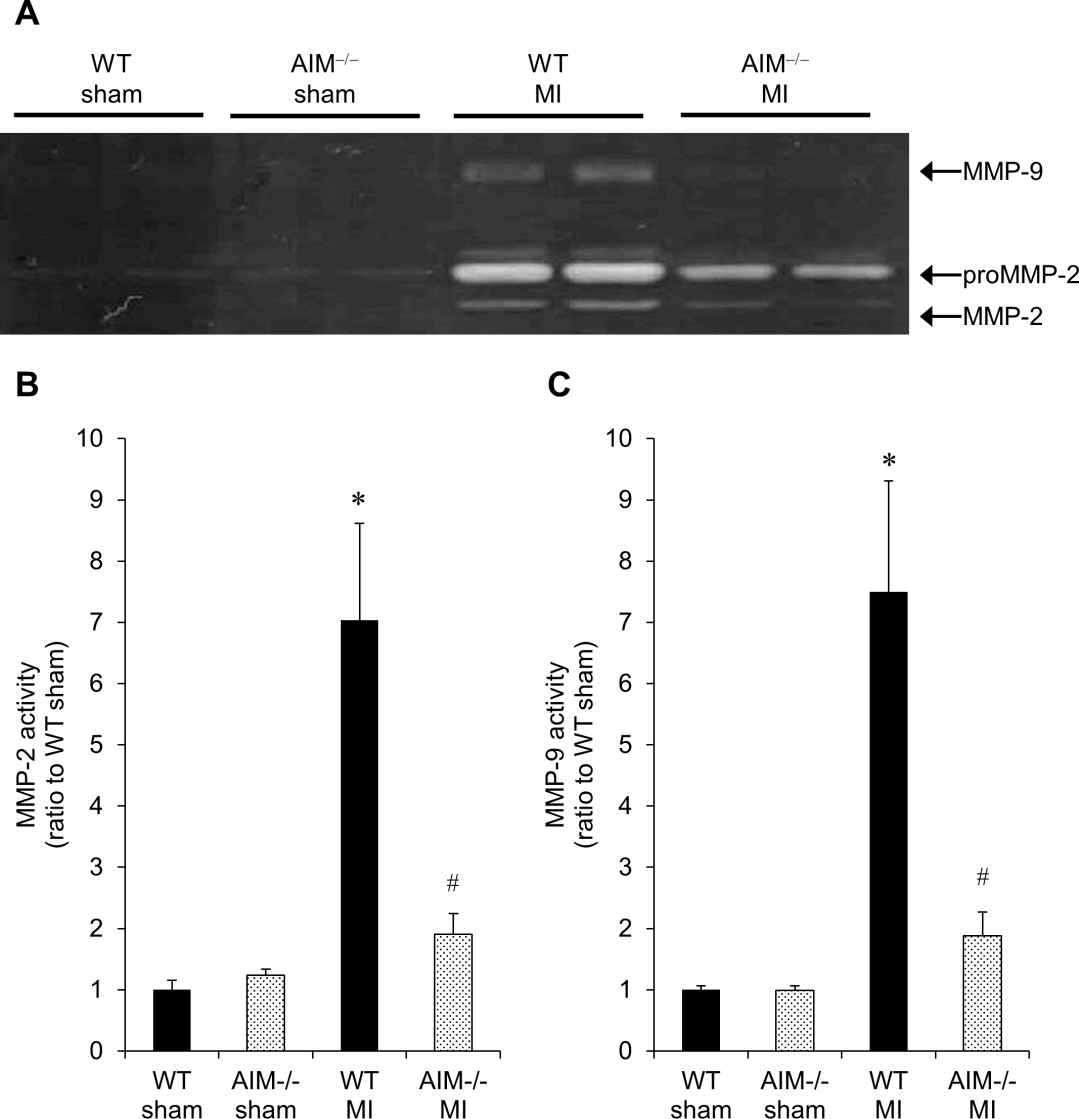
MMP-2 and 9 activity levels in the infarcted myocardium of WT and AIM^‒/‒^ mice at 7 days after MI. A representative image of gelatin zymography (**A**), and quantitative analyses of MMP-2 (**B**) and 9 (**C**) activity levels in the infarcted myocardium of WT and AIM^**‒/‒**^ mice at 7 days after MI. *P<0.05 compared with sham-operated WT mice, ^#^*P*<0.05 compared with WT-MI mice, n = 6 per group.

We measured MMP-2 and 9 activity levels by gelatin zymography. As shown in Fig 7A–7C, there were no significant differences in MMP-2 or 9 activity levels in the myocardium between sham-operated WT and AIM^**‒/‒**^ mice. In WT mice, both MMP-2 and MMP-9 activity levels in the infarcted myocardium were significantly higher than those in sham-operated myocardium. However, in AIM^**‒/‒**^ mice, there were no significant differences in MMP-2 or 9 activity levels between the infarcted and sham-operated myocardium. Both MMP-2 and MMP-9 activity levels in the infarcted myocardium of AIM^**‒/‒**^ mice were significantly lower than those of WT mice. We confirmed the molecular weight of MMP-2 and 9 by immunoblotting (S3 Fig).

## Discussion

In this study, the survival rate of AIM^**‒/‒**^ mice was significantly higher than that of WT mice in the acute phase after MI. Regarding the incidence of cardiac rupture, the rate of cardiac rupture was significantly lower in AIM^**‒/‒**^ mice than in WT mice. The infarct size determined by the morphometric analysis of picrosirius red stained left ventricular cross-sections at 7 days after MI and the parameters measured by echocardiographic and hemodynamic analysis at 3 or 7 days after MI were comparable. Immunohistology and RT-PCR analysis showed the number of M1 macrophages and the expression levels of M1 markers (iNOS and IL-6) in the infarcted myocardium at 3 days after MI were significantly lower in AIM^**‒/‒**^ mice than in WT mice. In contrast, there was no difference in the number of M2 macrophages and the expression of M2 markers (Arg-1, CD206 and TGF-β1) between the two groups. The ratio of apoptotic macrophages in total macrophages in the infarcted myocardium was significantly higher in AIM^**‒/‒**^ mice than in WT mice at 3 days after MI. MMP-2 and 9 activity levels in the infarcted myocardium were significantly lower in AIM^**‒/‒**^ mice than in WT mice at 7 days after MI.

AIM is a member of the scavenger receptor cysteine-rich superfamily [16]. It is secreted exclusively by macrophages and supports the survival of macrophages against various apoptosis-inducing stimuli [17, 18]. In addition to suppressing apoptosis, AIM promotes M1 monocyte/macrophage recruitment to inflamed tissues by facilitating MCP-1 expression [19, 20]. AIM contributes to macrophage accumulation in the inflamed tissue by maintaining survival and recruiting macrophages.

Although there are few studies about on cardiovascular disease, a recent study demonstrated that AIM contributed to the progression of adverse cardiac remodeling in the chronic phase after MI [31]. However, there is no study to reveal the role of AIM in the acute phase.

We demonstrated that AIM protein levels were increased after MI. Although, previous study reported the expression of AIM in the normal heart was very low [32], macrophages infiltrating into the infarcted myocardium might secrete AIM protein.

AIM depletion improved survival rates by reducing the incidence of cardiac rupture in the acute phase after MI. Although the risk factors for cardiac rupture are infarct size and blood pressure [33, 34], we demonstrated there was no difference in these parameters between WT and AIM^**‒/‒**^ mice.

It was reported macrophage accumulation in the infarcted myocardium involved in cardiac rupture after MI [12, 13]. Macrophages play a pivotal role in cardiac remodeling after MI. Macrophages are grouped into the following two phenotypes (M1 and M2), the main functions of M1 macrophages include phagocytosis of cellular debris at sites of myocardial damage, secretion of inflammatory cytokines and reorganization of tissue matrices by producing metalloproteinases (MMPs) in the acute phase after MI [10, 11]. In contrast, M2 macrophages facilitate resolution of inflammation and regeneration by promoting myofibroblast accumulation, collagen deposition, and angiogenesis [12].

Because macrophage infiltration in the infarcted myocardium is peaks at 3–7 days after MI [23, 35, 36, 37], we evaluated it at 3 and 7 days after MI. Our data showed that the numbers of M1 macrophages and the expression levels of M1 markers, such as iNOS and IL-6 [10, 38, 39, 40], but not M2 markers, such as Arg-1, CD206 and TGF-β1 [9, 10, 38, 39], in the infarcted myocardium after MI were significantly lower in AIM^**‒/‒**^ mice than in WT mice.

This phenomenon may be attributable to the following mechanism: AIM depletion may promote macrophage apoptosis in the infarcted myocardium, or suppress M1 macrophage recruitment by a decrease in MCP-1 expression.

In our study, the ratio of apoptotic macrophages in total macrophages in the infarcted myocardium was significantly higher in AIM^**‒/‒**^ mice than in WT mice at 3 days after MI, whereas there was no significant difference in the expression of MCP-1 in the infarcted myocardium at 3 days after MI between WT and AIM^**‒/‒**^ mice. These data suggested AIM depletion might suppress M1 macrophage accumulation in the infarcted myocardium through promoting macrophage apoptosis.

M1 macrophages are a potent source of MMP-2 and 9 in the acute phase after MI. MMP-9 is secreted mainly by neutrophils and M1 macrophages, and its activity peaks at 3 to 4 days after MI [35, 41, 42]. In contrast, MMP-2 is secreted mainly by fibroblasts and myofibroblasts, and its activity peaks at approximately 7 days after MI [35, 42]. However, M1 macrophages are also a potent source of MMP-2 [35, 43]. Although MMP-2 and 9 contribute to reorganization of tissue matrices in the acute phase after MI [10, 11], it has been suggested that excessive extracellular matrix degradation due to MMP activation may contribute to cardiac rupture after MI [38, 44].

In this study, the number of M1 macrophages and the activity levels of MMP-2 and 9 were significantly lower in AIM^**‒/‒**^ mice than in WT mice in the infarcted myocardium in the acute phase after MI, indicating that AIM depletion may suppress the activity of MMP-2 and 9 by decreasing the levels of M1 macrophages, which are a potent source of MMP-2 and 9.

## Conclusions

AIM depletion decreases the levels of M1 macrophages, which are a potent source of MMP-2 and 9, in the infarcted myocardium in the acute phase after MI by promoting macrophage apoptosis, and leads to a decrease in the incidence of cardiac rupture and improvements in survival rates.

## Supporting information

S1 Fig Immunoblotting for AIM in the infarcted myocardium of WT and AIM^‒/‒^ mice at 7 days after MI. (TIF)

S2 Fig Representative images of picrosirius red-stained cardiac tissue sections of WT and AIM^‒/‒^ mice at 7 days after MI. (TIF)

S3 Fig Representative images of zymography (A) and immunoblotting (B) for MMP-2 and 9 in the infarcted myocardium at 7 days after MI.
(TIF)
