# Supplementary figures and images for: Apoptosis inhibitor of macrophage depletion decreased M1 macrophage accumulation and the incidence of cardiac rupture after myocardial infarction in mice
Source: PLoS One. 2017 Nov 9;12(11):e0187894. doi: 10.1371/journal.pone.0187894 (PMC5679665; doi:10.1371/journal.pone.0187894)

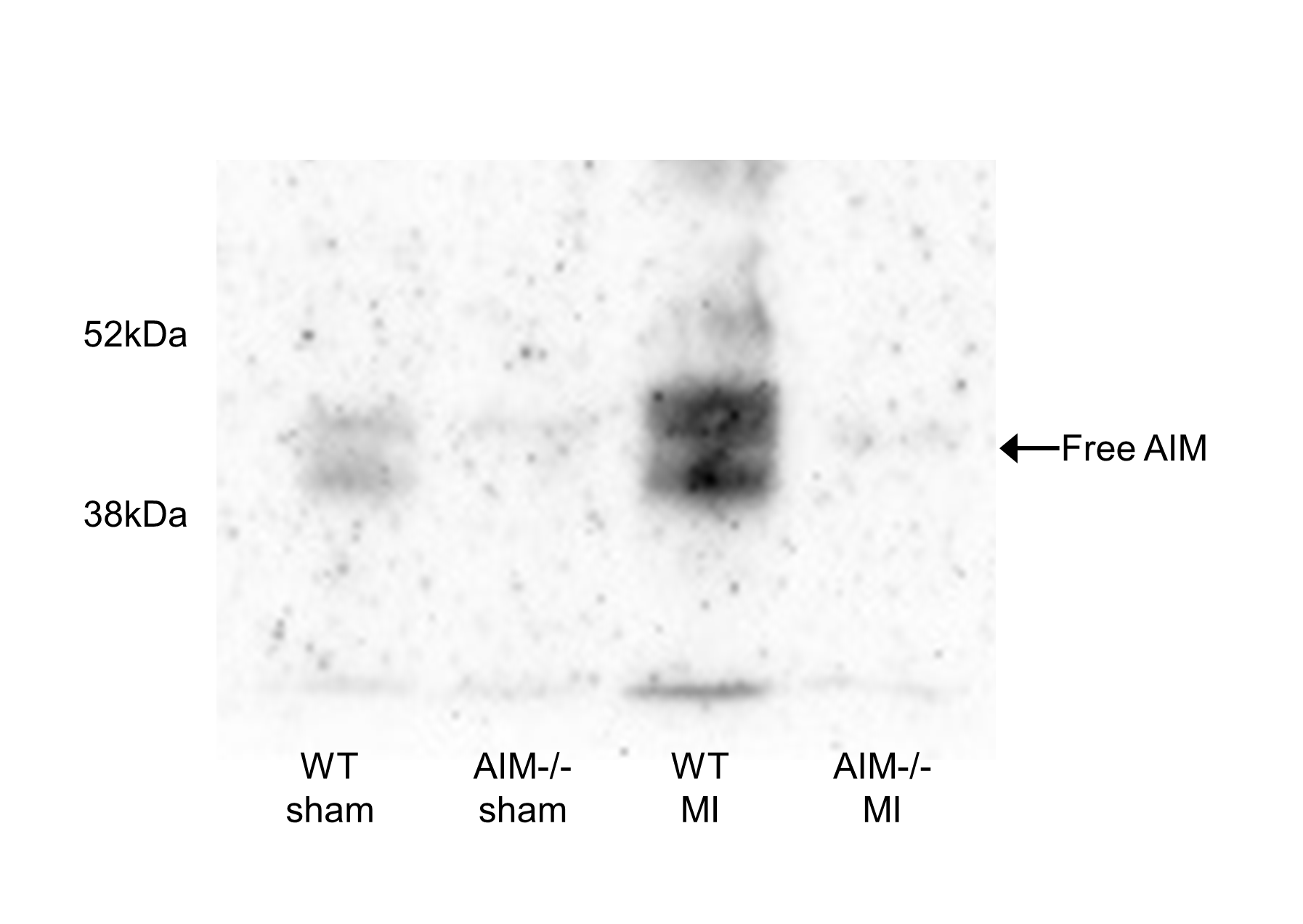

Supplement: S1 Fig — (TIF) [file pone.0187894.s001.tif]

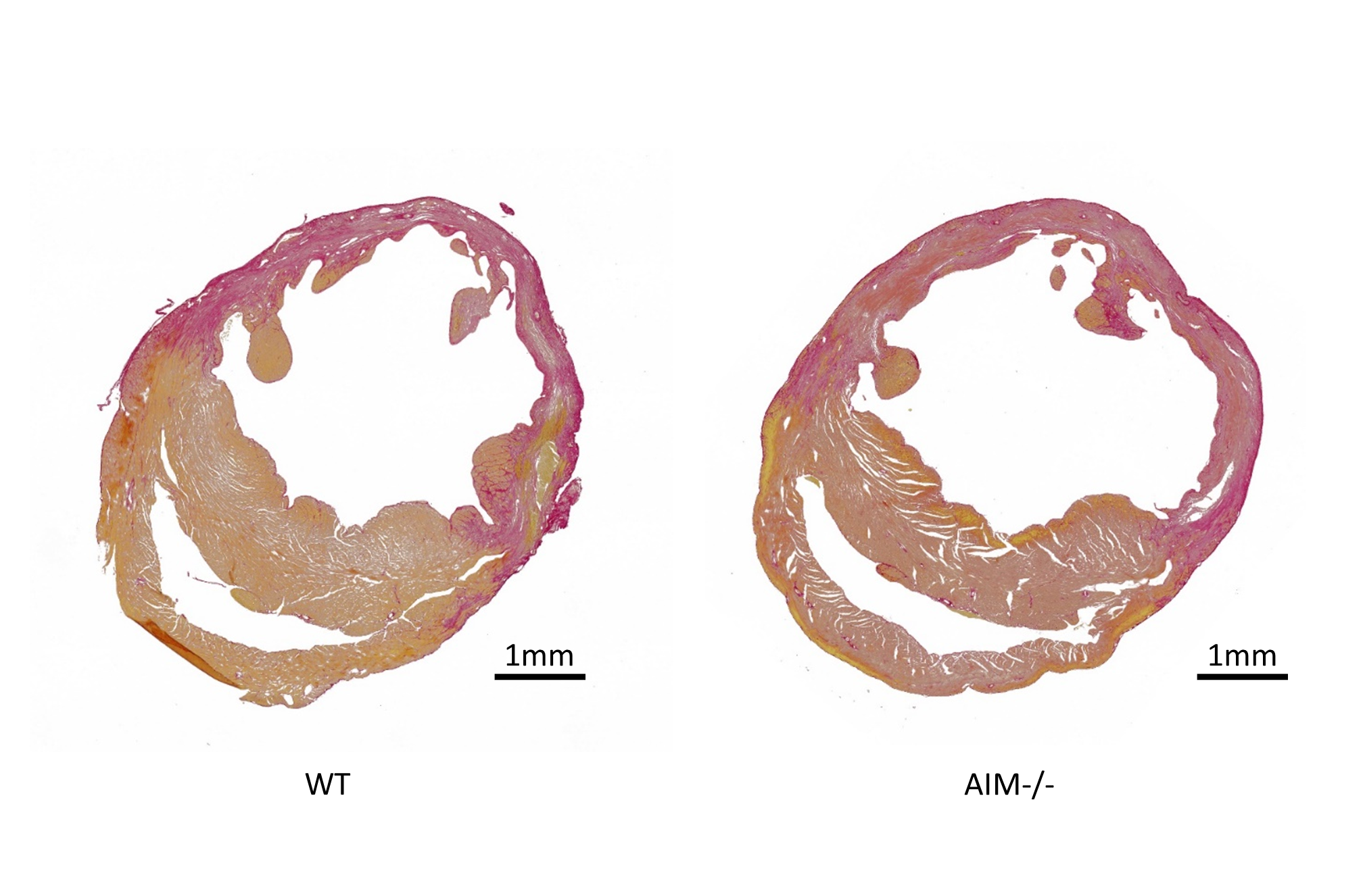

Supplement: S2 Fig — (TIF) [file pone.0187894.s002.tif]

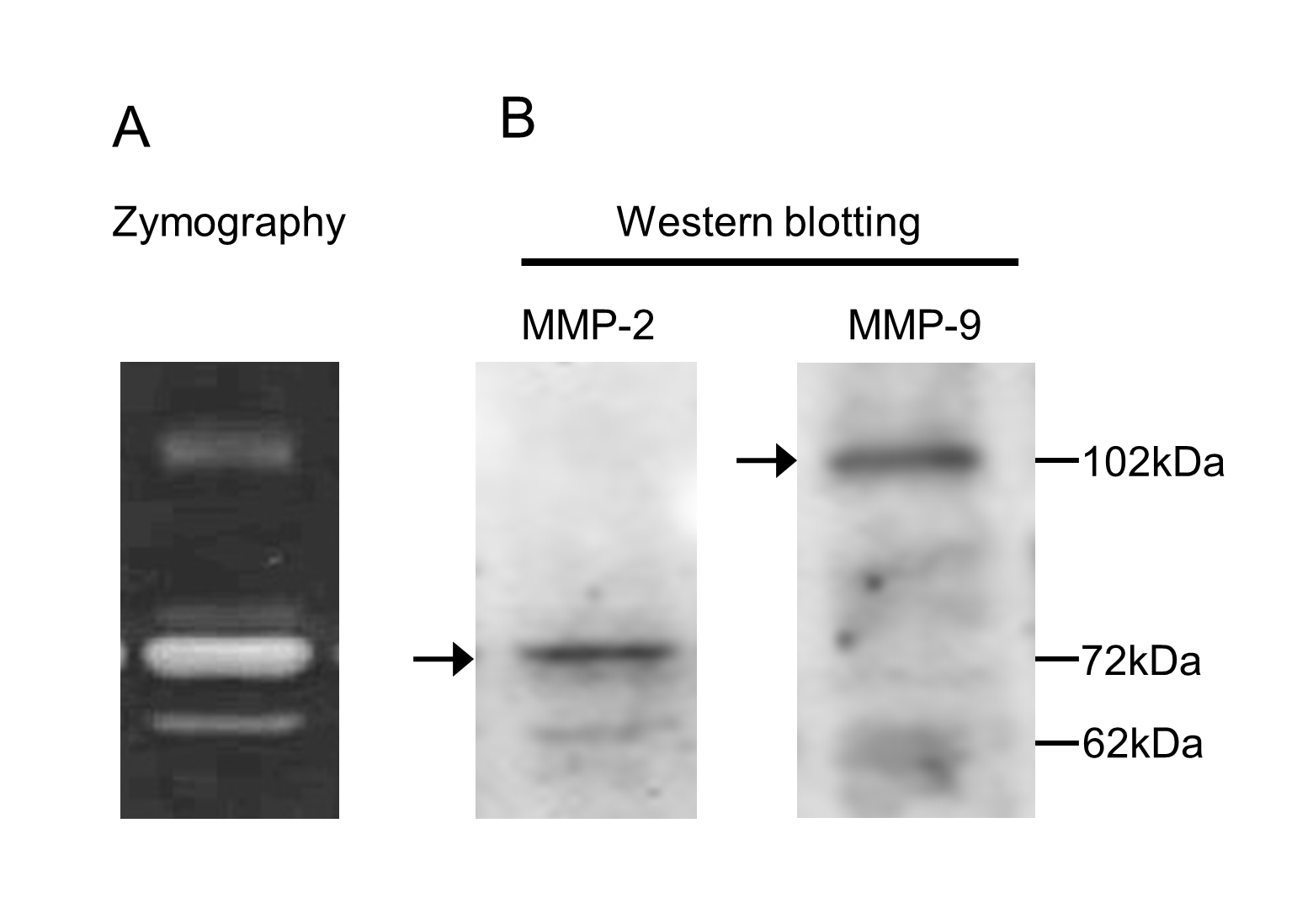

Supplement: S3 Fig — Representative images of zymography (A) and immunoblotting (B) for MMP-2 and 9 in the infarcted myocardium at 7 days after MI. (TIF) [file pone.0187894.s003.tif]
